# Home visits by community health workers in rural South Africa have a limited, but important impact on maternal and child health in the first two years of life

**DOI:** 10.1186/s12913-020-05436-7

**Published:** 2020-06-29

**Authors:** Linnea Stansert Katzen, Mark Tomlinson, Joan Christodoulou, Christina Laurenzi, Ingrid le Roux, Venetia Baker, Nokwanele Mbewu, Karl W. le Roux, Mary Jane Rotheram Borus

**Affiliations:** 1 Institute for Life Course Health Research, Department of Global Health, Faculty of Medicine and Health Sciences, Stellenbosch University, Office 4009, 4th Floor, Education Building, Francie van Zijl Drive, Tygerberg, South Africa; 2 School of Nursing and Midwifery, Queens University, Belfast, UK; 3 Department of Psychiatry and Biobehavioral Sciences, Semel Institute, University of California, 10920 Wilshire Blvd., Suite 350, Los Angeles, California 90024 USA; 4 Philani Maternal, Child Health and Nutrition Trust, Phaphani Street, Site C, Khayelitsha, Cape Town, South Africa; 5 Department of Family Medicine, Walter Sisulu University, Nelson Mandela Drive, Mthatha, 5117 South Africa; 6 Primary Health Care Directorate, Old Main Building, Groote Schuur Hospital, E47-25, Observatory 7925, Cape Town, South Africa; 7 Zithulele Training and Research Centre, Zithulele Hospital, Mqanduli District, Eastern Cape, South Africa

**Keywords:** Maternal depression, Community health workers, Maternal and child health, Breastfeeding, Rural health

## Abstract

**Background:**

More than 50% of Africa’s population lives in rural areas, which have few professional health workers. South Africa has adopted task shifting health care to Community Health Workers (CHWs) to achieve the Sustainable Development Goals, but little is known about CHWs’ efficacy in rural areas.

**Methods:**

In this longitudinal prospective cohort study, almost all mothers giving birth (*N* = 470) in the Zithulele Hospital catchment area of the OR Tambo District were recruited and repeatedly assessed for 2 years after birth with 84.7–96% follow-up rates. During the cohort assessment we found that some mothers had received standard antenatal and HIV care (SC) (*n* = 313 mothers), while others had received SC, supplemented with home-visiting by CHWs before and after birth (HV) (*n* = 157 mothers, 37 CHWs). These visits were unrelated to the cohort study. Multiple linear and logistic regressions evaluated maternal comorbidities, maternal caretaking, and child development outcomes over time.

**Results:**

Compared to mothers receiving SC, mothers who also received home visits by CHWs were more likely to attend the recommended four antenatal care visits, to exclusively breastfeed at 3 months, and were less likely to consult traditional healers at 3 months. Mothers in both groups were equally likely to secure the child grant, and infant growth and achievement of developmental milestones were similar over the first 2 years of life.

**Conclusion:**

CHW home visits resulted in better maternal caretaking, but did not have direct benefits for infants in the domains assessed. The South African Government is planning broad implementation of CHW programmes, and this study examines a comprehensive, home-visiting model in a rural region.

## Background

Globally, it is anticipated that there will not be sufficient professional health care providers to meet the need for care until at least 2050. While there are 24.2 physicians per 10,000 people in the United States, there are only 7.6 in South Africa [1]. This deficit is exaggerated in rural areas, where 43% of the South African population live [2], and where only 12% of doctors and 19% of nurses are based [3].

Although the South African government offers free primary health care, rural clinics are understaffed, and healthcare workers or medications may not always be available. In addition, patients face significant barriers to accessing rural healthcare facilities: transport is often unavailable; distances are vast; and geographical barriers such as rivers and mountains restrict mobility [4].

Given these challenges, health system administrators often focus on task shifting from professional to paraprofessional staff in order to meet the health needs of communities [5]. In 2011 the South African government launched a new policy on the re-engineering of primary health care that includes using teams of generalist community health workers (CHWs) [6]. However, there have been few studies evaluating whether these programmes are effective in impoverished rural areas that may make CHW intervention more difficult or present unique challenges. For maternal and child health in particular, there are many barriers that must be addressed by CHW.

The HIV prevalence in the area, 29.8% is similar to the national prevalence of 30% [7]. However, the actual effect of HIV in rural areas is often unknown as most research takes place in urban centres.

Within this already impoverished and difficult context, families are affected by multiple additional, potentially reversible, interacting challenges: maternal depression, alcohol use, and intimate partner violence (IPV). The rates of perinatal depression in studies conducted in South Africa have ranged from 27 to 47% [8–10]. Depressed mothers are less likely to be responsive to their infants, often retarding infant growth, resulting in behavioural problems, and reducing cognitive functioning [11–13].

Alcohol abuse is a common issue in South Africa. About 25% of mothers in South Africa drink alcohol and most of them report problematic drinking behaviours [9, 14], associated with negative child outcomes [15]. Alcohol use in pregnancy can have a lifelong effect on the baby, as it can lead to Fetal Alcohol Spectrum Disorders (FASD). South Africa has one of the highest rates of FASD in the world [16]. Further, intimate partner violence is often associated with alcohol abuse [14]. Women experiencing intimate partner violence both prior to and during pregnancy are at risk for multiple poor maternal and infant health outcomes, such as pre-term labour and infants with low birth weights [17].

Fortunately, previous CHW interventions have been shown to ameliorate these challenges. A comprehensive CHW home visiting program in Cape Town that offers mothers support on interrelated health issues and helps integrate them into existing health systems has had success addressing these issues [9, 18–20]. Mothers were more likely to complete important caretaking tasks for their children. Mothers living with HIV were more likely to adhere to guidelines to reduce and prevent mother-to-child transmission of HIV. Mothers receiving the CHW home visits breastfed longer, as recommended by the WHO, and their infants had better growth [9, 19, 21].

Other potentially protective factors, such as obtaining a child support grant (CSG), avoiding traditional healers, and attending a minimum of four antenatal care visits, have not yet been evaluated, but are crucial to improving maternal and child health in rural settings. Traditional healers, common in South Africa are often seen as a source of medical misinformation and dangerous practices, as well as reducing other avenues of maternal health care seeking and causing treatment delays [22, 23]. Furthermore, traditional healers often give herb mixtures to new-borns, more than half of infants before 1 month, and this interferes with exclusive breastfeeding and may at times be toxic [24].

For the roll-out of CHW to be successful in rural settings of South Africa, the ability to address multiple maternal co-morbidities and promote maternal caretaking that benefits healthy child development in a challenging setting is essential. Many CHW programs are ‘vertical’ interventions, focusing on one key issue such as HIV or TB in a targeted way. However, infants in South Africa face multiple challenges and therefore, a CHW program with a more comprehensive approach is implemented in this study.

This study in a deeply rural community in the Eastern Cape of South Africa aims to assess MCH over the first 2 years of life and compares outcomes for mothers and their babies living in areas with standard home antenatal and HIV care compared to those receiving standard care and home visits from a ‘horizontally’ integrated program. In this program, CHW provide comprehensive support on interrelated health issues and are integrated into the existing health systems [9, 18–20].

## Methods

This longitudinal prospective observational cohort study was conducted with approval of the Institutional Review Board of the Stellenbosch University (N12/08/046) and permission for the recruitment to occur in government health facilities was granted by the Eastern Cape Department of Health. All procedures performed in studies involving human participants were in accordance with the ethical standards of the institutional and/or national research committee and with the 1964 Helsinki declaration and its later amendments or comparable ethical standards. This article does not contain any studies with animals performed by any of the authors.

### Setting

Zithulele Hospital lies within the King Sabata Dalindyebo (KSD) Sub-district of the OR Tambo District of the Eastern Cape Province of South Africa. The Eastern Cape Province has some of the worst child health indicators in South Africa. The OR Tambo district, in particular, is one of the most under-developed and impoverished districts in the country [25]. Most households lack electricity, running water, and access to toilet facilities. The unemployment rate is well over 70% and the average annual income per household is R14 600 ($1518 in 2013) [26]. The hospital has a catchment area of nearly 1300 km^2^ and serves a population of 130,000–150,000 [26].

### Participants

From January through April 2013, a consecutive series of mothers giving birth at Zithulele Hospital and its 10 closest clinics, as well as mothers who had a home birth in the areas covered by the ten clinics, were approached to participate in a birth cohort study, the Zithulele Births Follow up Study (ZiBFUS). Mothers who gave birth at the hospital but lived outside this geographical catchment area were excluded. From 493 live births, 470 mothers provided written voluntary informed consent (4.7% refusal rate). In the case where the mother was less than 18 years old (16.4%), written consent was obtained from the adolescent mother and one of her parents/guardians. Written informed consent was obtained from all individual participants included in the study. In the cohort of 470 mother-infant pairs, we found that mothers living in certain villages in the hospital catchment area had received home visits by CHW of a non-profit organisation (NPO), the Philani Nutrition Trust, during the antenatal period and during their baby’s first 2 years of life. Mothers visited by these CHWs were labelled as being part of the home visiting (HV) group, while the others were designated as the standard care (SC) group. These home visits were unrelated and not part of the longitudinal prospective cohort study.

### Description of intervention

The Philani Maternal, Child Health and Nutrition Trust is a NPO operating in the catchment area of Zithulele Hospital in the Eastern Cape since 2010. Philani Mentor Mothers (CHWs) are recruited from the areas where they live and are trained to conduct home visits in their communities. The training includes a four-week course on maternal and child health, HIV and TB, followed by 2 weeks of in-the-field training and shadowing of experienced CHWs. There is a strong emphasis on careful recruitment, ongoing training, supportive in-the-field supervision and accountability [18].

CHW are assigned a catchment area and systematically conduct home visits in their designated area/village. CHW offer to weigh children to make sure they are growing well, as a way to gain entry into the household. While there, they provide health education about nutrition, breastfeeding, HIV, PMTCT, TB and the importance of antenatal care and find out if any of the women in the household are pregnant. Some pregnant mothers are found by word of mouth in the community. A file is opened for all pregnant mothers and for all children who are under-weight for age, stunted or wasted, and these households are paid careful attention to by the CHWs and regularly visited. The Philani CHW programme schedule requires that mothers are visited every month early in pregnancy, twice in the eighth month of pregnancy, weekly in the ninth month of pregnancy, within 2–3 days of arriving home after birth, weekly in the first month after birth and then every 2 weeks up until the infant is 1 year old and monthly after this.

### Assigning participants to standard care (SC) or home visiting (HV) groups

All mothers in both the Standard Care Group and the Home Visiting Group were recruited to the study in exactly the same way – 1-2 days post birth if delivering in hospital and within 2 weeks if delivering at home - by a research team completely independent of the CHW programme. 77% of mothers recruited gave birth at the hospital, 9% at a community health centre, 4% on the way to a health facility and 10% at home. To identify women who gave birth at home or in one of the feeder clinics/community health centers, the research team relied on nurses at the clinic to identify these cases and offered nurses a small airtime reimbursement to contact the field workers with the woman’s telephone number. The majority of home births were referred to us by nurses at the time of the mother’s first visit to the clinic, usually within a few days after birth. Field workers (known as data collectors) would then visit mothers at home. We believe that virtually all home births during the recruitment period were identified in this way because of the strong incentive for mothers to visit a clinic or hospital soon after birth to acquire a Road-to-Health card (RtHC) for their baby. Not only is the RtHC a type of health ‘passport’ and an extremely important health record, but it is also used to apply for a child support grant ($25 per month in 2013) from the South African Social Security Agency (SASSA).

Mothers were assigned to the Standard Care group (SC) or the Home visiting group (HV) at analysis, based on whether or not they indicated that they had been visited by a Philani Mentor Mother (CHW) antenatally or after delivery.

### Standard care group (SC)

All women recruited to the study have access to free primary health care, which includes antenatal care and HIV care, most often provided at clinics. At the time of the study, “Option A” Prevention of Mother-to-child-transmission (PMTCT) was in place, in which mothers living with HIV that have a CD 4 count of less than 350 are started on lifelong highly active antiretroviral therapy (HAART) and those with a CD4 of over 350 receive Zidovudine (AZT) from 14 weeks gestation and a stat dose of nevirapine (NVP) during childbirth, and infants of mothers living with HIV are given NVP syrup. All mothers living with HIV were encouraged to continue to monitor their CD4 count and to test their children for HIV at 6 weeks of age and again 6 weeks after the conclusion of breastfeeding.

### Home visiting group (HV)

Mothers in the HV group lived in areas where CHWs had been active in the community for at least 2 years, as described above and indicated that they had been visited by a Philani Mentor Mother (CHW).

### Assessments

A separate team of local women underwent extensive training as Data Collectors, with role-plays, certification, and random observations in the field over time. Interviewers recorded interviews and collected data on programmed mobile phones. When possible, information collected from the mothers was verified on mother’s antenatal clinic cards or children’s Road to Health Cards (RtHC). The data was also later reviewed with a supervisor and randomly audited. Mothers were interviewed and infants were assessed at six time points: during recruitment near the time of giving birth; at 3 months (85% follow-up, *n* = 390/460); at 6 months (92% follow-up, *n* = 420/456), at 9 months (88% follow-up, *n* = 410/454); at 12 months (91% follow-up, *n* = 411/450), and at 24 months (88% follow-up, *n* = 396/450). There were 22 infant deaths and all the second twins of the 9 sets of twins were not included in these analyses, to avoid double counting those families in the data set.

### Analysis

The primary analysis compared mothers receiving HV and mothers receiving SC using mixed effects regression models, with mothers receiving SC as the reference group. Logistic mixed effects regression models were used for binary outcomes. All models were adjusted for repeated measures, where appropriate, and a random participant effect was used to control for the longitudinal nature of the assessments. For all analyses, all observations that have a missing value for any one of the variables used in the model were excluded. All analyses were carried out using IBM SPSS Statistics (Version 20, Armonk, NY: IBM Corp).

### Demographic information

At baseline, mothers reported their age, education level, employment, their household make-up, household monthly income, access to a water source, and electricity. In addition, they reported on previous pregnancies and HIV status. HIV status was confirmed in the mother’s antenatal clinic card and the RtHC and compared to the Zithulele Hospital maternity statistics.

### Reversible maternal comorbidities

Depression was reported using the Edinburgh Postnatal Depression Scale (EPDS) [27]. We report both the mean scale score and identify mothers whose responses indicate probable depressive disorder using a cut-off of EPDS > 13 to indicate depressed mood [28]. Alcohol use prior to and during pregnancy was self-reported by the mothers. IPV was self-reported prior to pregnancy and during pregnancy.

### Protective maternal caretaking activities

Mothers who completed at least four antenatal care visits, by report and confirmed on the antenatal card, were reported. Mothers who tested for HIV during pregnancy, by report and confirmed in antenatal clinical card, were reported. Of mothers living with HIV, we report on four PMTCT tasks: 1) taking appropriate ART before birth; 2) giving the child nevirapine as appropriate; 3) 6-weeks post-delivery PCR test done; and 4) whether PCR was done by 6-months post-delivery. PCR status was both self-reported and verified by interviewers assessing the RtHC. The number of months of exclusive breastfeeding and those completing 6 months of breastfeeding was self-reported by mothers. Immunisations were recorded at each assessment as documented in the child’s RtHC. With informed consent, photographs were also taken of the immunisation page in the RtHC to ensure accuracy. We report on whether all required immunisations were complete at each interview. Mothers that secured a child support grant at each follow-up assessment were documented. Finally, mothers self-reported visiting traditional healers in the last 3 months.

### Child growth and development

Low birth weight was recorded for those infants < 2500 g. Infant growth was measured at each assessment. Children’s weight and height measures were then converted to Z-scores based on the WHO age-adjusted norms [29]. Growth is reflected in standardized scores (Z-scores) for height-for-age (HAZ), weight-for-age (WAZ), and weight-for-height (WHZ). A Z-score below − 2 SD was considered a serious health deficit: either stunting (> − 2SD for HAZ), wasting (> − 2 for WHZ) or severe underweight for age (> − 2 SD WAZ) [30]. The gross motor developmental milestones of the WHO for children at 6 (WHO1), 9 (WHO3), 12 (WHO1–5) and 24 months (WHO1–6) were administered [31, 32]. These include six milestones that are fundamental to acquiring self-sufficient locomotion: sitting without support, hands-and-knees crawling, standing with assistance, walking with assistance, standing alone, and walking alone [33] Each task was scored (0) if unable to perform, (1) if reported able, but not demonstrated, and (2) if demonstrated able to perform. A single developmental score was calculated by taking the mean of all the WHO Gross Motor Developmental milestone tasks [33].

## Results

The demographic characteristics of the mothers receiving SC (67%; *n* = 313) and HV (33%, *n* = 157) are summarized in Table 1. In both groups, mothers were about 25 years of age; however, adolescents were less likely to be in HV than SC (10% vs. 20%, *OR* = 0.44, *95% C.I.* = 0.24, 0.80, *p* = .007). Mothers had about an eighth-grade education and 4% were employed. Only about one-third of mothers lived with their family of origin. Households averaged about six persons each and about half of households (46%) had monthly incomes over R5000 (US$520). Only 16% had water on the premises. Mothers in HV were less likely to have electricity than mothers in SC (4% vs. 21%, *OR* = 0.15, *95% C.I.* = 0.06, 0.35, *p* < .001). About 40% of mothers were having their first baby. Across the region 29% of mothers were mothers living with HIV; however, mothers living with HIV were more likely to be in the HV than the SC group (35% vs. 26%, *OR* = 1.53, *95% C.I.* = 1.01, 2.31, *p* = .045).

**Table 1:** Baseline demographics

	Standard care (SC) *N* = 313	Home visiting (HV) *N* = 157	All mothers *N* = 470
Demographic characteristics			
Mean age (SD)	24.9 (7.6)	24.8 (6.3)	24.9 (7.2)
Adolescents*	20%	10%	16%
Mean highest education level (SD)	8.6 (2.3)	8.8 (2.6)	8.6 (2.4)
Employed	4%	3%	4%
Live with father or in-laws	34%	33%	33%
Number of people in household (SD)	5.9 (2.8)	5.8 (3.3)	5.87 (2.9)
Monthly household income >R2000 (US$208 in 2013)	48%	50%	48%
Water tank on site	18%	13%	16%
Electricity**	21%	4%	16%
Maternal health			
Primipara	40%	38%	40%
HIV Seropositive*	26%	35%	29%

Note. *P*-values based on mixed linear (continuous) and logistic (binary) regressions using random intercepts for mothers

* *p* < .05; ** *p* < .01

Maternal reversible comorbidities and protective caretaking activities over time are summarized in Table 2. Overall, 42% of mothers experienced one of these potentially reversible comorbidities. There was a suggested trend for mothers in the HV group to show less depressive symptoms over time compared to SC, this effect is however not statistically significant (Estimate – 0.55, Standard Error – 0.33, p-0.98). Alcohol use was similar over time in both groups. Fewer mothers reported alcohol use after learning about their pregnancy (11% vs. 7%, *Estimate* = −.05, *Std. Error* = .01, *p* = .001). Overall, 27% of mothers reported IPV. HV mothers were more likely to report intimate partner violence (IPV) than SC mothers during pregnancy (29% vs 15%, *OR* = 1.78, *95% C.I.* = 1.13, 2.78, *p* = .012). IPV is more common during pregnancy, compared to when the mothers are not pregnant (*Estimate* = 0.11, *Std. Error* = .02, *p* < .001). Mothers in HV were more likely to complete their four antenatal visits prior to delivery than mothers in SC (38% vs. 26%, *OR* = 1.85, *95% C.I.* = 1.14, 3.00, *p* = .012). In both groups, 94% of mothers were tested for HIV during their pregnancy. Of the Mothers living with HIV, 5% were taking ART before the birth of their child, and 93% reported giving their child nevirapine post-birth. The HIV PCR testing rates for infants of mothers living with HIV were similar in both groups at 29% at 3 months and 48% at 6 months. Mothers receiving HV were more likely to breastfeed exclusively at 3 months compared to SC (29% vs. 17%, *OR* = 1.97, *95% C.I.* = 1.20, 3.25 *p* = .008). Completion of immunizations on time over the first 24 months of life was also similar in both groups. While immunization rates were low for the 6-, 10- and 14-week immunizations (49% at 3 months), many mothers “caught up” with these immunizations by 12 months post-birth (73%). The time to get the CSG was similar in both groups. Only about 38% of mothers started receiving a child grant by 3 months post-birth. By 1 year, the majority of mothers received the child grant (about 77%) and 90% received the child grant by 24 months post-birth. Significantly fewer mothers receiving HV had taken their infants to a traditional healer at 3 months (6% vs.13% OR = 2.33, 95%C.I. = 1.04, 5.20, *p* = .039), compared to mothers receiving SC.

**Table 2:** Reversible maternal comorbidities and protective caretaking activities in home visiting (HV) and standard care (SC) groups over time

	Standard care (SC)	Home visiting (HV)	All mothers
	*N* = 313	*N* = 157	*N* = 470
	% n	% n	% n
*Reversible maternal comorbidities*			
Maternal depression (EPDS > 13)			
At birth	15%	17%	16%
3 months post-birth	15% (37/241)	15% (19/124)	15% (56/365)
6 months post-birth	15% (37/250)	12% (13/112)	14% (50/362)
9 months post-birth	12% (27/223)	11% (13/117)	12% (40/340)
12 months post-birth	16% (33/209)	9% (9/103)	13% (42/312)
24 months post-birth	8% (14/177)	6% (5/85)	7% (19/262)
Alcohol use			
Before learning of pregnancy	11%	12%	11%**
After learning of pregnancy	6%	8%	7%**
Intimate partner violence			
Prior to pregnancy	9%	15%	11%**
During pregnancy*	19%	29%	22%**
*Protective maternal caretaking activities*			
During pregnancy			
Four or more ANC visits*	26%	38%	48%
Tested for HIV	93%	94%	94%
PMTCT tasks for mothers living with HIV			
ART before birth	3%	7%	5%
NVP at birth	91%	96%	93%
PCR testing, 3 months	24%	36%	29%
PCR testing, 6 months	51%	43%	48%
Feeding			
Exclusive breastfeeding, 3 months**	17%	29%	21%
Exclusive breastfeeding, 6 months	18% (47/264)	25% (32/126)	20% (79/390)
Immunization			
Up-to-date at 3 months	51%	45%	49%
Up-to-date at 6 months	72%	75%	73%
Up-to-date at 9 months	84%	83%	84%
Up-to-date at 12 months	77%	66%	73%
Child support grant (CSG)			
Secured by 6 months	63%	71%	66%
Secured by 12 months	76%	80%	77%
Traditional healers			
Visited within 3 months*	13%	6%	10%

Note. *P*-values based on linear mixed (continuous) and logistic (binary) regressions that account for repeated measures with random intercepts for mothers

* *p* < .05; ** *p* < .01

Child growth and development measures are measured in Table 3. About 25% of all children had low birth weights (less than 2500 g). In both groups at birth, the growth Z-scores for weight for age and for weight for height were about 0.5 SD lower than WHO norms. Children experienced similar growth over time on HAZ, WAZ, and WHZ. In both groups, WAZ (*Estimates* = [0.48–0.80], *Std. Error* = [0.06–0.08]) and WHZ (*Estimates* = [0.31–1.01], *Std. Error* = [0.09–0.10]) increased significantly from birth through the first 2 years of life (*p*’*s* < .001). In both groups, HAZ increased from birth at 3 (*Estimate* = 0.44, *Std. Error* = .08, *p* < .001), 6 (*Estimate* = 0.26, *Std. Error* = .09, *p* = .004), and 9 (*Estimate* = 0.19, *Std. Error* = .09, *p* = .04) months post-birth. HAZ were not significantly different from birth at 12 or 24 months. The completion of the WHO developmental milestones at 12 and 24 months was similar in both groups.

**Table 3:** Child growth and development outcomes in home visiting (HV) and standard care (SC) groups over time

	Group	0	3	6	9	12	24
Low birth weight	SC	26%	–	–	–	–	–
	HV	22%	–	–	–	–	–
Stunting (HAZ < -2 SD)	SC	7%	3%	7%	7%	7%	7%
	HV	7%	4%	4%	6%	10%	9%
Severe low-weight for age (WAZ < -2 SD)	SC	11%	5%	7%	2%	4%	4%
	HV	7%	3%	2%	5%	4%	2%
Wasting (WHZ < -2 SD)	SC	19%	10%	5%	4%	0%	3%
	HV	16%	9%	4%	1%	3%	5%
Mean WHO development score (SD)	SC	–	–	1.20 (0.41)	1.98 (0.13)	1.94 (0.30)	1.98 (0.22)
	HV	–	–	1.36 (0.50)	1.97 (0.17)	1.96 (0.19)	2.00 (0.00)

## Discussion

This study found important associations between mothers receiving home visits (HV) by CHWs and protective maternal caretaking tasks. Mothers receiving HV were significantly more likely to make the four recommended antenatal care visits [34] and were more likely to be exclusively breastfeeding at 3 months. They were also less likely to have taken their infants to a traditional healer at 3 months, potentially protecting their infants from harmful practises. These are important improvements in maternal caretaking, especially in rural areas.

The increased utilization of antenatal care by mothers living in HV areas is not unexpected. A key message given by CHW is that mothers should regularly visit PHC clinics for antenatal care. It is reassuring that this exhortation by CHW appears to be resulting in measurable action from mothers living in the HV areas.

In this cohort, reversible maternal comorbidities play an important role. We found that 42% of mothers were dealing with depression, alcohol use, or intimate partner violence. Depressive symptoms for all mothers decreased after delivery. The findings suggested a trend of fewer depressive symptoms among mothers receiving HV when compared to mothers receiving SC, however this effect was not statistically significant. Given the higher percentage of mothers living with HIV in the HV group, we would not expect this trend. These results are consistent with findings from an urban setting in South Africa where, at 36 months post birth, mothers receiving home visits showed significantly fewer depressive symptoms than mothers who did not receive home visits [19].

By chance, the mothers receiving HV were more likely to be Mothers living with HIV (MLH), to be adolescent mothers, and to have less electricity than those receiving SC. MLH are typically more depressed than peers [35] and have many more tasks to complete while pregnant and a new mother. Adolescent mothers are typically worse caretakers and are more likely to experience IPV [17]. A lack of electricity indicates that the area may have had access to fewer economic or structural resources. Having electricity also conveys economic and health benefits. Given these initial differences, both maternal and child outcomes would be expected to be worse for mothers receiving HV than those receiving SC.

There was no significant difference in HIV testing, for Mothers living with HIV to complete PMTCT, to complete immunizations, or to receive the child grant. The delay in obtaining the child grant is concerning, as the grant is of vital importance in rural areas where the majority of households rely on grants for household income [36]. It is concerning that the rate of PCR tests completed at in the first 6 months is so low (29% at 3 months and 46% at 6 months). There were no differences in child outcomes over time in the two groups. Child growth was similar. The children were typical in reaching their development milestones.

We found alcohol use was similar in both groups for women prior to pregnancy recognition, 11%, and similarly lower during pregnancy, 7%. This appears substantially lower than the 25% reported in the peri-urban townships around Cape Town [9, 14] . The lower rates of alcohol consumption in the rural areas may be linked to a more traditional lifestyle, in which drinking is not as accepted as in urban areas, particularly for women. While we do not believe this is a major contribution to the numbers reported here, there is also concern about potential under-reporting of alcohol consumption [37]. Still, as the WHO recommends that women abstain from drinking alcohol during pregnancy, the finding that 7% of the women studied drank whilst aware of their pregnancy, is of concern.

In our study, Intimate Partner Violence was not only prevalent (27% of all mothers over time), but more mothers reported IPV during pregnancy compared to IPV prior to pregnancy. In addition, more mothers receiving HV reported experiencing violence than mothers receiving SC. This may be due to higher reporting of IPV amongst HV mothers as a result of trust built through their relationship with their CHW. With this finding, CHW interventions that target pregnant women should consider incorporating messages about IPV.

In the OR Tambo District of South Africa’s rural Eastern Cape, there have been significant improvements in both maternal and child outcomes over the last 10 years [38], yet substantial needs remain. This study builds on a series of evaluations of peri-urban home-visiting in Khayelitsha, Cape Town, South Africa [9, 18, 19, 39] by examining maternal and child outcomes in mothers receiving home visits or not in a deeply rural setting.

Implementation of the program through an established organization is an important feature in developing a successful intervention. The Philani intervention programme has been operating in peri-urban townships outside of Cape Town since the 1990s, and was expanded to the Eastern Cape 3 years prior to this study. With this specific area, the OR Tambo district of the Eastern Cape, a variety of factors, such as poverty, limited resources and limited access to healthcare [25, 38] make it more difficult for these organizations to thrive. Yet, even soon after being implemented, this programme is significantly associated with improved maternal caretaking.

Although receiving HV is associated with significant effects, improvements can be made to increase the impact of the HV. There were larger differences in more domains and child outcomes were improved when mounting this programme in the peri-urban townships [9, 18, 19]. More impact of the intervention was expected, indicating that there is room for improvement in adapting this program to a deeply rural setting. Effective program design and management, acceptability of the program in communities served and securing support from political leaders or other health care professionals have been identified as crucial parts in expanding CHW programs [40]. More research into possible gaps in these crucial domains is needed to determine reasons for limited results in this study. Migration is a possible reason for the limited results; migration in rural South Africa is common, meaning that it is possible that mothers have been out of the CHW area for parts of the study time, hence only receiving limited intervention*.* In addition, we did not monitor or manage how regularly visits occurred as this study was done independently of the CHW programme. Thus, data about the number of home visits and time points for these visits were not collected, and their impact was not considered in this study, but this is an important area for future research. A different set of challenges face home visiting programs in rural areas, and practical and logistical considerations—such as transport over large distances, access to transport and resources, must be central concerns in program design and implementation [6, 39]. Among more social and structural dimensions of health—such as interpersonal violence and alcohol usage—there were no significant effects seen from the CHW intervention at this stage, and CHW training must likely be adapted to address these behaviours. This study showed that CHW home visiting had limited, albeit important effects on maternal and child health, the results highlights the importance of tailoring intervention to contexts, in this case a deeply rural area.

### Limitations

This study was not a randomized controlled trial. Outcomes were evaluated on an intention-to-treat basis. We did not examine how the number of home visits may be related to outcomes. The majority of maternal comorbidities are self-reported by the mothers; we would have desired more biomarkers. However, maternal ANC visits, children’s health visits, growth, and the CSG are validated on the RTHC. In the future, a randomized controlled trial is needed to evaluate this program.

## Conclusions

Home-visiting by CHWs in rural South Africa benefits MCH by increasing maternal caretaking – seen in increased antenatal care visits, exclusive breastfeeding for longer, and using traditional healers less often. There are areas where improvements in CHWs training are needed. More attention to structural and social dimensions of health may be necessary for more comprehensive maternal and child programming and should be a focus for future research.

## Data Availability

The datasets generated and/or analysed during the current study are available from the corresponding author upon reasonable request.

## Abbreviations

ANC: Antenatal Care
CHW: Community Health Worker
CSG: Child Support Grant
EPDS: Edinburgh Postnatal Depression Scale
FASD: Fetal Alcohol Spectrum Disorders
HIV: *Human Immunodeficiency Virus*
HV: Home Visiting
IPV: Intimate Partner Violence
MCH: Maternal and Child Health
MLH: Mothers living with HIV
PMTCT: Prevention of Mother to Child Transmission
RtHC: Road to Health Card
SC: Standard Care

## References

[1] Campbell J, Dussault G, Buchan J, Pozo-Martin F, Guerra Arias M, Leone C, et al. A universal truth: No health without a workforce. 2013. doi: ISBN 978 92 4 150676 2.

[2] Rural Health Advocacy Project (2015). Rural Health Fact Sheet 2015.

[3] Hamilton K, Yau J. The Global Tug-of-War for Health Care Workers. Migr Inf Source. 2004:1–9. http://www.migrationpolicy.org/article/global-tug-war-health-care-workers.

[4] Harris B, Goudge J, Ataguba JE, McIntyre D, Nxumalo N, Jikwana S (2011). Inequities in access to health care in South Africa. J Public Health Policy.

[5] Singh P, Sachs JD (2013). 1 million community health workers in sub-Saharan Africa by 2015. Lancet..

[6] Austin-Evelyn K, Rabkin M, MacHeka T, Mutiti A, Mwansa-Kambafwile J, Dlamini T (2017). Community health worker perspectives on a new primary health care initiative in the eastern cape of South Africa. PLoS ONE.

[7] National Department of Health. The 2012 National Antenatal Sentinel HIV & Herpes Simplex type-2 Prevalence Survey, South Africa, National Department of Health. Pretoria: National Department of Health; 2013. p. 1–86. www.health-e.org.za/wp.../ASHIVHerp_Report2014_22May2014.pdf.

[8] Hartley M, Tomlinson M, Greco E, Comulada WS, Stewart J, le Roux I, et al. Depressed mood in pregnancy: Prevalence and correlates in two Cape Town peri-urban settlements. Reprod Health. 2011:9. 10.1186/1742-4755-8-9. 10.1186/1742-4755-8-9 PMC3113332 21535876

[9] Le Roux I, Tomlinson M, Harwood JM, O’Connor MJ, Worthman CM, Mbewu N (2013). Outcomes of home visits for pregnant mothers and their infants: a cluster randomized controlled trial. Aids..

[10] Rochat TJ, Tomlinson M, Bärnighausen T, Newell M, Stein A (2011). The prevalence and clinical presentation of antenatal depression in rural South Africa. J Affect Disord.

[11] Parsons CE, Young KS, Rochat TJ, Kringelbach ML, Stein A (2012). Postnatal depression and its effects on child development: a review of evidence from low- and middle-income countries. Br Med Bull.

[12] Rahman A, Malik A, Sikander S, Roberts C, Creed F (2008). Cognitive behaviour therapy-based intervention by community health workers for mothers with depression and their infants in rural Pakistan: a cluster-randomised controlled trial. Lancet..

[13] Stewart RC (2007). Maternal depression and infant growth - a review of recent evidence. Matern Child Nutr.

[14] Davis EC, Rotheram-Borus MJ, Weichle TW, Rezai R, Tomlinson M (2017). Patterns of alcohol abuse, depression, and intimate partner violence among township mothers in South Africa over 5 years. AIDS Behav.

[15] Chen J-H (2012). Maternal alcohol use during pregnancy, birth weight and early behavioral outcomes. Alcohol Alcohol.

[16] May PA, Blankenship J, Marais AS, Gossage JP, Kalberg WO, Barnard R (2013). Approaching the prevalence of the full Spectrum of fetal alcohol Spectrum disorders in a south African population-based study. Alcohol Clin Exp Res.

[17] Silverman JG, Decker MR, Reed E, Raj A (2006). Intimate partner violence victimization prior to and during pregnancy among women residing in 26 U.S. states: associations with maternal and neonatal health. Am J Obstet Gynecol.

[18] Rotheram-Borus MJ, le Roux IM, Tomlinson M, Mbewu N, Comulada WS, le Roux K (2011). Philani plus (+): a Mentor mother community health worker home visiting program to improve maternal and infants’ outcomes. Prev Sci.

[19] Tomlinson M, Rotheram-Borus MJ, le Roux IM, Youssef M, Nelson SH, Scheffler A (2016). Thirty-six-month outcomes of a generalist paraprofessional perinatal home visiting intervention in South Africa on maternal health and child health and development. Prev Sci.

[20] Tulenko K, Møgedal S, Afzal MM, Frymus D, Oshin A, Pate M (2013). Community health workers for universal health-care coverage: from fragmentation to synergy. Bull World Health Organ.

[21] Rotheram-Borus MJ, Richter LM, Van Heerden A, Van Rooyen H, Tomlinson M, Harwood JM, et al. A cluster randomized controlled trial evaluating the efficacy of peer mentors to support south African women living with HIV and their infants. PLoS ONE. 2014;9(1):e84867. 10.1371/journal.pone.0084867 PMC3898948 24465444

[22] Bland R, Rollins N, Van Den Broeck J, Coovadia H& CHG (2004). The use of non-prescribed medication in the first 3 months of life in rural South Africa. Trop Med Int Health.

[23] Peltzer K, Phaswana-Mafuya N, Treger L (2009). Use of traditional and complementary health practices in prenatal, delivery and postnatal care in the context of HIV transmission from mother to child (PMTCT) in the eastern cape, South Africa. Afr J Tradit Complement Altern Med.

[24] Sibeko L, Dhansay MA, Charlton KE, Johns T, Gray-donald K (2005). Beliefs , Attitudes , and Practices of Breastfeeding Mothers From a Periurban Community in South Africa. J Hum Lact.

[25] Gaunt B (2010). Are we winning? Improving perinatal outcomes at a deeply rural district hospital in South Africa. South African Med J.

[26] Statistics South Africa. Census 2011 - Census in brief. 2012. doi: ISBN 978–0–621-41388-5.

[27] Cox JL, Holden JM, Sagovsky R (1987). Detection of Postnatal Depression: Development of the 10-item Edinburgh Postnatal Depression scale. Br J Psychiatry.

[28] Rochat TJ, Tomlinson M, Newell M-L, Stein A (2013). Detection of antenatal depression in rural HIV-affected populations with short and ultrashort versions of the Edinburgh postnatal depression scale (EPDS). Arch Womens Ment Health.

[29] World Health Organization, United Nations Childrens Fund. WHO child growth standards and the identification of severe acute malnutrition in infants and children. 2009. doi:http://www.who.int/nutrition/publications/severemalnutrition/9789241598163/en/. 24809116

[30] World Health Organization (2010). Interpretation Guide Nutrition Landscape Information System (NLIS).

[31] Wijnhoven TMA, De Onis M, Onyango AW, Wang T, Bjoerneboe GA, Bhandari N (2004). Assessment of gross motor development in the WHO multicentre growth reference study. Food Nutr Bull.

[32] Lansdown RG, Goldstein H, Shah PM, Orley JH, Di G, Kaul KK (1996). Culturally appropriate measures for monitoring child development at family and community level : a WHO collaborative study. Bull World Health Organ.

[33] de Onis M, WHO Motor Development Study. Windows of achievement for six gross motor development milestones. Acta Peadiatrica. 2006;450:86–95. 10.1111/j.1651-2227.2006.tb02379.x 16817682

[34] Villar J, Ba’aqeel H, Piaggio G, Lumbiganon P, Belizán JM, Farnot U (2001). WHO antenatal care randomised trial for the evaluation of a new model of routine antenatal care. Lancet..

[35] Dyer TP, Stein JA, Rice E, Rotheram-Borus MJ (2012). Predicting depression in mothers with and without HIV: the role of social support and family dynamics. AIDS Behav.

[36] Vaaltein S, Schiller U (2017). Addressing multi-dimensional child poverty: the experiences of caregivers in the eastern cape, South Africa. Child Youth Serv Rev.

[37] Del Boca FK, Darkes J (2003). The validity of self-reports of alcohol consumption: state of the science and challenges for research. Addiction..

[38] le Roux K, Akin-Olugbade O, Katzen LS, Laurenzi C, Mercer N, Tomlinson M (2017). Immunisation coverage in the rural eastern cape - are we getting the basics of primary care right? Results from a longitudinal prospective cohort study. South African Med J.

[39] le Roux IM, le Roux K, Mbeutu K, Comulada WS, Desmond KA, Rotheram-Borus MJ (2011). A randomized controlled trial of home visits by neighborhood mentor mothers to improve children’s nutrition in South Africa. Vulnerable Child Youth Stud.

[40] Pallas SW, Minhas D, Pérez-Escamilla R, Taylor L, Curry L, Bradley EH (2013). Community health workers in low- and middle-income countries: what do we know about scaling up and sustainability?. Am J Public Health.

[41] Stansert Katzen L. Paraprofessional home-visiting improves maternal caregiving for mothers living with and without HIV over the first two years of life in rural South Africa. In: the International AIDS Conference 2018 Abstract Book (conference proceedings on the internet); 2018 July 23–27; Amsterdam 2018 (cited 2020 June 15). p 1013. Available from: AIDS 2018. http://www.aids2018.org/Portals/4/File/AIDS2018_Abstract_book.pdf?ver=2018-08-06-160624-427.

